# Transcriptional Responses of *Beauveria bassiana* Blastospores Cultured Under Varying Glucose Concentrations

**DOI:** 10.3389/fcimb.2021.644372

**Published:** 2021-03-24

**Authors:** Gabriel Moura Mascarin, Natasha Sant’Anna Iwanicki, Jose Luis Ramirez, Ítalo Delalibera, Christopher A. Dunlap

**Affiliations:** ^1^ Laboratory of Environmental Microbiology, Brazilian Agricultural Research Corporation, Embrapa Environment, Jaguariúna, Brazil; ^2^ Department of Entomology and Acarology, “Luiz de Queiroz” College of Agriculture/University of São Paulo (ESALQ/USP), Piracicaba, Brazil; ^3^ Crop Bioprotection Research Unit, National Center for Agricultural Utilization Research, United States Department of Agriculture, Agriculture Research Service, Peoria, IL, United States

**Keywords:** biocontrol, dimorphic growth, morphogenesis, Cordycipitaceae, liquid fermentation

## Abstract

Culturing the entomopathogenic fungus, *Beauveria bassiana*, under high glucose concentrations coupled with high aeration results in a fungal developmental shift from hyphal growth to mostly blastospores (yeast-like cells). The underlying molecular mechanisms involved in this shift remain elusive. A systematic transcriptome analysis of the differential gene expression was preformed to uncover the fungal transcriptomic response to osmotic and oxidative stresses associated with the resulting high blastospore yield. Differential gene expression was compared under moderate (10% w/v) and high (20% w/v) glucose concentrations daily for three days. The RNAseq-based transcriptomic results depicted a higher proportion of downregulated genes when the fungus was grown under 20% glucose than 10%. Additional experiments explored a broader glucose range (4, 8, 12, 16, 20% w/v) with phenotype assessment and qRT-PCR transcript abundance measurements of selected genes. Antioxidant, calcium transport, conidiation, and osmosensor-related genes were highly upregulated in higher glucose titers (16-20%) compared to growth in lower glucose (4-6%) concentrations. The class 1 hydrophobin gene (*Hyd1*) was highly expressed throughout the culturing. *Hyd1* is known to be involved in spore coat rodlet layer assembly, and indicates that blastospores or another cell type containing hydrophobin 1 is expressed in the haemocoel during the infection process. Furthermore, we found implications of the HOG signaling pathway with upregulation of homologous genes *Ssk2* and *Hog1* for all fermentation time points under hyperosmotic medium (20% glucose). These findings expand our knowledge of the molecular mechanisms behind blastospore development and may help facilitate large-scale industrial production of *B. bassiana* blastospores for pest control applications.

## Introduction

The arthropod-pathogenic fungus *Beauveria bassiana* (Ascomycota: Cordycipitaceae) is globally used as a biological control agent against many crop pests and vectors of human diseases. Moreover, this fungus has been recently identified as an endophyte associated with various benefits including plant growth, drought resistance, and induced disease resistance (Mascarin et al., 2016; Tomilova et al., 2020). The current use of *B. bassiana*, as well as other filamentous (anamorphic phase) entomopathogenic fungi, as biopesticides have been primarily focused on the inundative biocontrol approach, *i.e*. large quantities of fungal inoculum are produced then deployed in the field to deliver a lethal dose to the target pest, aiming to achieve quick and effective pest suppression (Jaronski, 2010). Therefore, to meet the growing demands of high-quality large-scale production of fungal propagules for the biopesticide industry, liquid culture may be the preferred method over solid-state fermentation. Liquid culture production technology provides various advantages in terms of easiness, automated operation, scalability, quality control, batch-to-batch uniformity, and cost-effective, large-scale production afforded by deep-tank stirred bioreactors for some entomopathogenic fungi like *B. bassiana* (Jaronski, 2010; Mascarin et al., 2015a; Mascarin et al., 2015b; Mascarin et al., 2016).

As many Hypocrealean insect-pathogenic fungi, dimorphic growth in *B. bassiana* is ubiquitous. It renders unicellular vegetative, thin-walled, yeast-like cells termed blastospores, which can be easily mass-produced *in vitro* by submerged liquid fermentation (Pham et al., 2009; Mascarin et al., 2015a; Mascarin et al., 2015b). These *in vitro* yeast-like cells possess distinct biochemical properties and surface structures from hemolymph-derived hyphal bodies (Wanchoo et al., 2009; Yang et al., 2017). This cellular phenotype evolved to facilitate fungal infection in arthropod hosts by successfully allowing the fungus to bypass the host immune system while simultaneously colonizing and exploiting host tissues and resources leading to nutrient depletion, tissue degradation by enzymes, and synthesis of secondary metabolites (Humber, 2008). For pest control purposes, blastospores can be formulated as sprayable products and outperform in virulence to the traditional solid-grown conidia against various target pests because this cell type can germinate quicker and infect the host sooner (Cliquet and Jackson, 1999; Kirkland et al., 2004a; Kirkland et al., 2004b; Mascarin et al., 2015a; Mascarin et al., 2015b; Alkhaibari et al., 2016; Alkhaibari et al., 2018; Bernardo et al., 2018; Bernardo et al., 2020). Besides that, blastospores’ virulence can be markedly affected by culture conditions and media composition against arthropod targets (Kirkland et al., 2004b; Mascarin et al., 2015a). Based on our previous studies, we have found a cost-effective technology for rapidly producing high yields of blastospores with low mycelium content by submerged liquid fermentation under high glucose titers and aeration rates (Mascarin et al., 2015a; Mascarin et al., 2015b; Mascarin et al., 2016). This phenomenon has intrigued us and has prompted investigation on the molecular mechanisms governing this improved phenotypic response during dimorphic growth, resulting in high production yields of blastospores in *B. bassiana* induced by greater glucose titer gradient coupled with high aeration supply.

Growth media for entomopathogenic fungi generally use up to 4% glucose as the carbon source to support conidia or blastospore production in solid or liquid media. A low carbon rate usually renders high mycelial growth and low blastospore yields (Mascarin et al., 2015a). However, our previous studies have shown proper C:N ratio coupled with high aeration rate and glucose titers induce higher blastospore yield with low hyphal growth in liquid culture of *B. bassiana* (Mascarin et al., 2015a; Mascarin et al., 2016). The mechanism behind this phenotype remains elusive but a plausible scenario falls under the hypothesis that oxidative and osmotic stress could be involved as part of the fungal response to such growth conditions.

Therefore, this study aimed at understanding the transcriptional signatures involved in blastospore formation and multiplication in response to the hyperosmolarity caused by a high gradient of glucose titer. This study also provides novel insights on osmotic and oxidative stress genes involved in the regulatory system of blastospore development in *B. bassiana* during submerged fermentation. In this regard, expression profiles of key genes were characterized across glucose gradients from 4% to 20%. This study reveals high osmotic pressure (> 0.5 MPa) induces improved blastospores’ production in *B. bassiana* and other related insect-pathogenic fungi. Thus, this study may help improve the scale-up industrial production of blastospores in other filamentous entomopathogenic fungi, including those recalcitrant isolates which often provide low yields.

## Methods

### Fungal Isolate and Culture Conditions

The *B. bassiana* strain GHA (ARSEF 201) selected for RNA sequencing was initially isolated from infected *Diabrotica undecimpunctata* in a greenhouse in Corvallis, Oregon, USA, in October of 1977. This strain is the active ingredient in several commercial mycoinsecticides. The isolate was cultured on potato dextrose agar (PDA) and preserved as a glycerol stock (10%) at -80 °C at the USDA, Peoria, IL. The culture conditions used for RNA-seq were selected because of high blastospore yields (Mascarin et al., 2015a; Mascarin et al., 2015b). The experiment consisted of seven treatments represented by an increased glucose gradient of 0, 4, 8, 10, 12, 16, and 20% glucose (w/v). Blastospore concentrations were recorded 24, 48, and 72 h post-inoculation of the fermentation course. Media composition consisted of 2.5% cottonseed flour (Pharmamedia^®^, ADM™, USA) as the nitrogen source, amended with basal salts and vitamin mix (Mascarin et al., 2015a). The cultures were placed on a rotary shaker incubator (New Brunswick™, Innova 4000^®^, NJ, U.S.A.) with rotating orbit diameter of 19 mm and agitation speed of 350 rpm, at 28°C, and a filling volume of 50 mL liquid medium in 250-mL baffled Erlenmeyer flasks to provide high aeration, as previously reported by (Mascarin et al., 2015a). Liquid media were inoculated with a pre-culture to deliver a final concentration of 5 × 10^6^ blastospores/mL. The experiment consisted of three biological replicates and was repeated twice or thrice on different dates using new fungal inoculum. The fungal growth parameters evaluated during the course of fermentation were: a) growth kinetics to determine the blastospore concentration from 24 h to 72 h post-inoculation; b) maximum specific growth rate determined during the log-growth phase of blastospore production curve at different glucose titers using the following formula: µ (h^-1^) = ln(N_i_) – ln(N_0_)/(t_i_ – t_0_), where N_i_ = blastospore population recorded in log-growth at time t_i_, N_0_ = initial blastospore population at time zero t_0_ (i.e., 5 × 10^6^ blastospores/mL); and c) glucose utilization by subtracting the difference between initial glucose titer and the remaining glucose measured in the spent medium at the end of the fermentation period (day 3).

Blastospore concentration data were fitted to a generalized linear model with gamma distribution and inverse link function, including glucose titer, fermentation day, and interaction term as fixed effects in the linear predictor. Specific growth rate and glucose consumption datasets were separately fitted to general linear models with and without a random effect for replicate flasks, and normal distribution for errors including glucose titer as the fixed effect. Multiple pairwise post-hoc comparisons using the Tukey HSD method (*p*-value < 0.05) were used to separate significant means between glucose titers within each fermentation day. This analysis was performed in the statistical environment R (version 4.0.2, R Core Team, 2020) and plots created with the “ggplot2” package (Wickham, 2016).

### RNA Isolation and Sequencing

Three independent biological samples, corresponding to 1 mL of the whole culture in shake flasks, were collected for each fermentation time (*i.e.*, days 1, 2, and 3). Samples were centrifuged at 16,000*g* for five minutes to pellet the cells, the supernatant was decanted, and the pellet was flash-frozen in liquid nitrogen. The pellet was homogenized with a mortar and pestle under liquid nitrogen. RNA was extracted with a Qiagen RNAeasy Kit (Germantown, MD, USA). RNA quality was confirmed using an Agilent 2200 tapestation. RNA concentration was measured using the Qubit^®^ RNA Assay Kit in Qubit^®^ 2.0 Fluorometer (Life Technologies, CA, USA). RNA integrity was assessed using the RNA Nano 6000 Assay Kit of the Agilent Bioanalyzer 2100 system (Agilent Technologies, CA, USA). RNA integrity number (RIN) was considered acceptable if greater than 6. The library preparation and sequencing were performed by the Brigham Young University DNA sequencing center. The mRNA was selected with an oligo poly dT selection, and the library was prepared with an Illumina TruSeq RNA library kit. The final library was sequenced with an Illumina HiSeq 2500 with 50 cycle single read sequencing V4. The transcriptome data are available on NCBI-GEO under the GEO accession GSE163673 and can be accessed through the link: https://www.ncbi.nlm.nih.gov/geo/query/acc.cgi?acc=GSE163673.

### RNA-Seq Data Analysis

Raw sequencing reads were checked for adapters and quality trimmed to Q30 using Genomics Workbench (version 10.0.1) (Qiagen, Inc, Germantown, MD, USA). The reads were mapped to the *B. bassiana* strain D1-5 reference genome (GenBank accession no. ANFO01000000) using the 85% length alignment and 85% similarity. Expression values were normalized using RPKM and a separate General Linear Model with a negative binomial distribution under Genomics Workbench. Statistical comparisons of differential gene expression between treatments were conducted pairwise with Wald tests and Bonferroni corrected *p*-values determined under Genomics Workbench. Differentially expressed genes with False Discovery Rate (FDR) adjusted *p*-value (< 0.005) and log2(fold change, FC) > 2 or < -2 were used for the final data set.

### Gene-Set Enrichment Analysis

The Gene Set Enrichment Analysis (GSEA) software determines whether *a priori* defined set of genes is statistically significant between two biological states (Subramanian et al., 2005). GSEA rank gene sets by enrichment magnitude and indicates classes of over-represented genes in the gene set. As recommended for RNA-seq datasets, GSEA was used in the GSEAPreranked mode with a user-provided list of all genes pre-ranked according to a defined metric: the log_2_ fold change, adjusted *p*-value, or inverse *p*-value, and a list of gene sets. GSEAPreranked calculates an enrichment score by matching genes from gene sets to those in the user ranked index. Next, the gene set enrichment score shows how often members of that gene set occur at the top or bottom of the ranked data set. This study used GSEAPreranked mode with gene sets categorized by Gene Ontology (GO) annotation. The metric used in the GSEA input file was the sign of fold change multiplied by its inverse *p*-value (Iwanicki et al., 2020a). The *p*-value provided was used as an output of differential gene expression analysis. When the *p*-value output was “0”, the “0” value was replaced by artificially high or low values “+1E+308” or “-1E+308” for up and down-regulated genes according to the sign of fold change. The parameter adopted for running the GSEAPreranked for GO were: minlengh 10 and maxlengh 500, enrichment statistic: “classic” and FDR-correction for multiple testing < 0.25 for enriched gene sets (Iwanicki et al., 2020a).

### Expression of Heat Shock Protein and Key Genes Associated With Response to Stress Conditions and High Osmolarity Glycerol Pathway

Heat shock proteins (HSPs) are a large superfamily of intracellular, molecular chaperones with molecular sizes ranging from less than 30 to ~100 kD and are involved in maintaining protein homeostasis in several physiological processes including response to stress conditions. A recent study has investigated the role heat shock proteins (Hsp70) have in virulence, cell wall integrity, antioxidant activity, and stress tolerance of *B. bassiana* strain ARSEF 2860 (Wang et al., 2020). This study explored gene expression of 14 Hsp70 homologs identified by Wang et al. (2020) in blastospores cultivated with high (20%) and moderate (10%) glucose amended liquid media with focus on those related to antioxidant activity and stress tolerance. In addition, gene expression of four Mitogen-activated protein kinases (MAPK) homologs: *Ste11* (BBAD15_g9298), *Ssk2* (BBAD15_g1898), *Pbs2* (BBAD15_g4699), *Hog1* (BBAD15_g6467) associated with HOG pathway in *B. bassiana* ARSEF 2860, and one homolog associated to multiple stresses were also explored (Wsc1, BBAD15_g1986) obtained from Liu et al. (2017) and Tong et al., (2019). In addition, we examined the expression profile of another set of select *B. bassiana* homologous genes involved in osmotic stress, including *CnA1* (BBAD15_g8778), *Ecm33* (BBAD15_g9622), *Ras1* (BBAD15_g11654), *Mkk1* (BBAD15_g4332), *pmr1* (BBAD15_g2615), *Gpcr* (BBAD15_g8874), *Cdk1* (BBAD15_g6748), *Mtd* (BBAD15_g10510), *Ssk1* (BBAD15_g8860), *Bmh1* (BBAD15_g2365), *Ohmm* (BBAD15_g7953), *Ktr4* (BBAD15_g4067), and *Mbf1* (BBAD15_g10650) (Ortiz-Urquiza and Keyhani, 2016). Sequences were blasted against the genome of *B. bassiana* D1-5 to obtain homologous genes (*e*-value < 0.0001 and sequence similarity > 80%). An interactive heatmap was drawn to illustrate the selected genes on days 1, 2, and 3. This analysis was performed in the statistical environment R (version 4.0.2, R Core Team, 2020) and heatmap created with the “ggplot2” package (Wickham, 2016) using the mean log2-FC values of gene expression patterns at 1, 2, and 3 days post-inoculation across the increased glucose gradient. Four selected genes (*Pbs2*, *Ste11*, *Hog1*, and *Ssk2*), known to be associated with response to osmotic stress were mapped against MAPK-signaling pathways by searching against the Kyoto Encyclopedia of Genes and Genomes pathway (KEGG) database (https://www.kegg.jp/kegg/tool/map_pathway.html). The mapping was performed through the K numbers of genes, while the pathways were shown as a schematic illustration.

### qRT-PCR-Based Gene Expression Analysis of Key Blastospore-Related Genes

In this experiment, *B. bassiana* GHA was cultivated with different glucose concentrations ranging from 4% to 20% at incremental intervals of 4%. The blastospore concentration, glucose consumption, and specific growth rate of blastospores were recorded daily for three days under the same set of fermentation conditions described previously. This bioassay’s primary purpose was to evaluate the expression of key genes involved in blastospore development across a greater glucose titer gradient. Samples stored at -80°C were used for RNA extraction *via* TRIzol reagent (Invitrogen^®^), and the concentration and quality of total RNA were evaluated *via* Nanodrop (Thermo Scientific). Synthesis of cDNA was conducted with 1ug of total RNA from each sample using the QuantiTec reverse transcription kit with DNA Wipeout (Qiagen). Measures of gene expression were performed in a 10 µL reaction using one microliter of cDNA and *B. bassiana* GHA gene-specific primers (**Supplemental Table S1**). Primers were designed on loci from *B. bassiana* strain D1-5 reference genome (GenBank accession no. ANFO01000000), which were updated with read mapping of *B. bassiana* GHA reads and a new consensus extracted. The PowerUp SYBR green Master mix qPCR kit (Qiagen) was used in all reactions using the qPCR cycling condition recommended by the manufacturer (holding at 95°C for 10 min, 40 cycles of 15 s at 95°C, and 1 min at 60°C). The qPCR assays included a Melt Curve analysis at the end of the reaction, and was carried out on an Applied Biosystems QuantStudio 6 Flex Real-time PCR system (ThermoFisher Scientific™). The gene expression assays evaluated at least three biological replicates, and each sample was assessed in duplicate. The gene expression levels were normalized against the fungal gene actin previously validated in Zhou et al. (2012) (**Supplemental Table S1**) and were analyzed post-run using the ΔΔCt method (Livak and Schmittgen, 2001). The temporal mean expression levels in fold change of selected *B. bassiana* genes during different blastospore growth stages under liquid fermentation were described in line plots, and an asterisk indicates significant differences between glucose titers at 5% probability level by ANOVA within each growth interval. The heatmap was created with the “ggplot2” package using the mean log_2_ fold change values of gene expression patterns at 1, 2, and 3 days post-inoculation across the increased glucose gradient.

## Results

We have previously argued blastospore production of *B. bassiana* requires a combination of high aeration and high osmotic pressure imposed by glucose to achieve high yields in shorter fermentation times. In our model system designed in this study, we set out transcriptome analysis to scrutinize the molecular and genetic factors driving blastospore formation in high glucose cultures (20% w/v) under high aeration rates against moderate glucose cultures (10% w/v) during three days of culture.

### Blastospore Yield and Kinetics Under Different Glucose Concentrations

**Figure 1:**
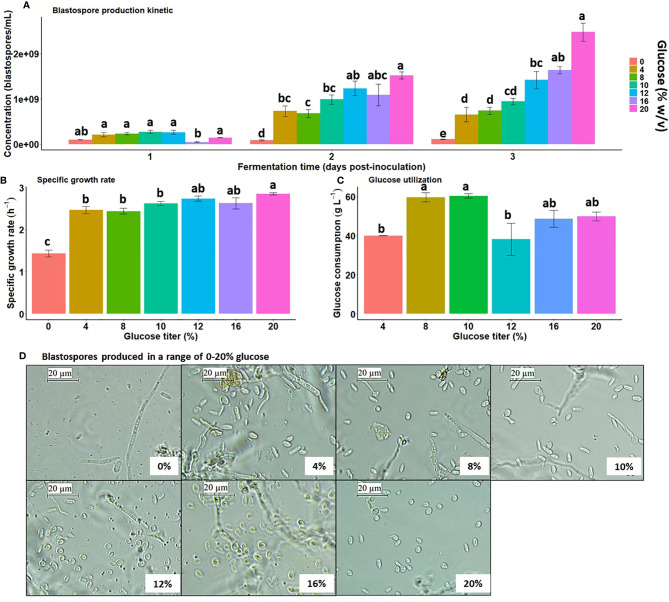
Blastospore yield and growth kinetics are significantly boosted by greater glucose gradient (0 to 20% w/v) under high aeration during submerged liquid culture conditions. **(A)** *B. bassiana* (strain GHA) blastospore concentration in liquid culture with a greater glucose titer gradient at 28 °C, 350 rpm, and 50 mL of filling volume. **(B)** Maximum specific growth rate during log-phase growth across glucose titer gradient. **(C)** Glucose consumption at day 3 post-inoculation across glucose titer gradient. Bar heights represent means, and error bars are standard error of means ( ± S.E.). **(D)** Blastospore morphotypes induced by different glucose titers (photographs taken at day 3 of fermentation). Vertical bars within each fermentation time are significantly different when followed by distinct letters (Tukey HSD, *p* < 0.05).

Liquid cultures of *B. bassiana* amended with varying glucose titers started with an initial density of 5×10^6^ blastospores/mL and dramatically increased over time with greater glucose gradient (Interaction day × glucose titer: $\chi_{12;79}^{2}=53.15$, *p* < 0.0001), indicating blastospore concentration is strongly dependent on fermentation time and glucose titer in the liquid medium (**Figure 1A**). Regardless of glucose titers tested, blastospore concentration at day 1 post-inoculation suggests fungal culture is under lag-phase adaptation to medium conditions. The lack of glucose in the medium resulted in steady and low blastospore yields over time. Notably, these fungal cultures exhibited more mycelium development than blastospores, suggesting this carbon source is indispensable to sustain the fungus yeast-like growth. From day 2 to day 3 post-inoculation, blastospore yields were always below 1×10^9^ blastospores/mL when grown in the range of 4-10% glucose with no significant blastospore concentration changes between titers. Blastospore production increased with increasing glucose concentration and peaked when grown with 20% glucose, yielding higher than 1×10^9^ blastospores/mL at 2 days post-inoculation.

The specific growth rate during the log-growth phase within the interval 0 and 2 days post-inoculation, was significantly increased with greater glucose titers (*F*_6;36_ = 22.76, *p* < 0.0001), reaching a plateau at 12% glucose and remained constant at 16% and 20% glucose (2.63 – 2.85 h^-1^). The specific growth rate at 20% glucose was greater than 0-10% glucose (**Figure 1B**). Glucose utilization by GHA cultures was significantly affected by the initial glucose titer (*F*_5;28_ = 4.66, *p* = 0.003), but the consumption trend did not increase with the glucose gradient. The maximum glucose uptake was around 60 g L^-1^ when cultures were grown under 8-10% initial glucose titers (**Figure 1C**). Moreover, the fungus’s glucose uptake remained lower than 50 g L^-1^ (= 5% w/v) when cultured with 12-20% glucose, indicating a surplus of spent glucose in the medium. Morphological examination of blastospores grown under greater glucose titers from 12% to 20% exhibited more ovoid shape and a smaller size than those produced with a range of 0-10% glucose (**Figure 1D**).

Considering the main objective to study blastospore growth, the glucose concentrations used in the transcriptome analysis (20% *versus* 10%) were chosen to avoid the interference of hyphal growth. Although there were no statistical differences among specific growth rates in media ranging from 4% to 10% glucose, fungal cultures in 10% glucose presented reduced hyphal growth compared to lower glucose concentrations (Mascarin et al., 2015a). Hence, 10% glucose was selected as the lower glucose titer to compare with the higher, 20% glucose, in the transcriptome study.

### Summary of RNAseq Data

A total of 108.56 million clean and mapped RNA-Seq reads were generated. Of these, 103.7 million reads could be aligned to the *B. bassiana* strain D1-5 reference genome (concordant unique pairs, see details in **Table 1**). A total of 54.3 and 53.8 million unique match reads were obtained for blastospores grown in medium amended with either 10% or 20% glucose. The average rate of reads alignment against the reference genome were 95.1% and 96.3% for 10% and 20% glucose treatments. **Table 1** summarizes the RNA-Seq experiment results, and transcriptomics data are available on NCBI-GEO under the GEO accession GSE163673.

**Table 1:** Summary of the read-mapping statistics on the transcriptomic features of *B. bassiana* liquid cultures grown over time.

Glucose level	10%			20%		
Fermentation time	Day 1	Day 2	Day 3	Day 1	Day 2	Day 3
Total mapped reads (%)	18,266,154 (95.88%)	23,776,977 (94.95%)	12,471,404 (94.44%)	21,119,505 (96.19%)	17,892,329 (95.89%)	15,029,519 (96.96%)
Total unmapped reads (%)	785,841 (4.12%)	1,265,577 (5.05%)	734,891 (5.56%)	836,200 (3.81%)	766,802 (4.11%)	471,220 (3.04%)
Unique match (%)	18,212,828 (95.59%)	23,699,923 (94.64%)	12,433,499 (94.15%)	21,071,725 (95.97%)	17,837,883 (95.60%)	14,9860,077 (96.68%)

**Figure 2:**
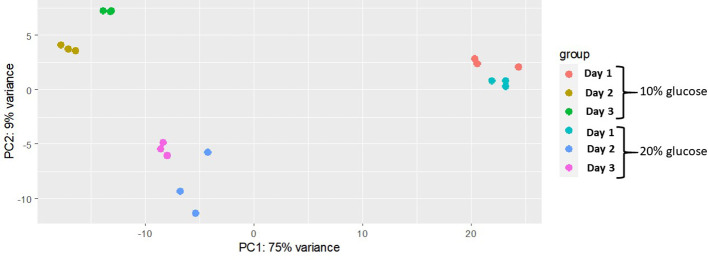
Multivariate analysis separating glucose treatments across different fermentation time points. Principal component analysis of regularized-logarithmic (rlog) transformed gene counts of *B. bassiana* (strain GHA) blastospores cultivated in medium with 10% or 20% glucose during three days of growth. Biological replicates are represented by each dot (n = 3 per treatment).

The principal component analysis showed the highest variation in the gene expression profile is from biological samples from days 1, 2 and 3 (75%), regardless of glucose concentration. According to the second principal component (accounted for 9% total variance), genes were differently expressed in blastospores grown with moderate (10%) glucose in relation to blastospores grown high glucose cultures (20%) at days 2 and 3 of fermentation. In general, biological samples were grouped within treatments indicating high similarity (**Figure 2**).

### Comparisons Between 20% and 10% Glucose-Amended Medium Within Days of Cultivation

#### Gene-Set Enrichment Analysis

**Figure 3:**
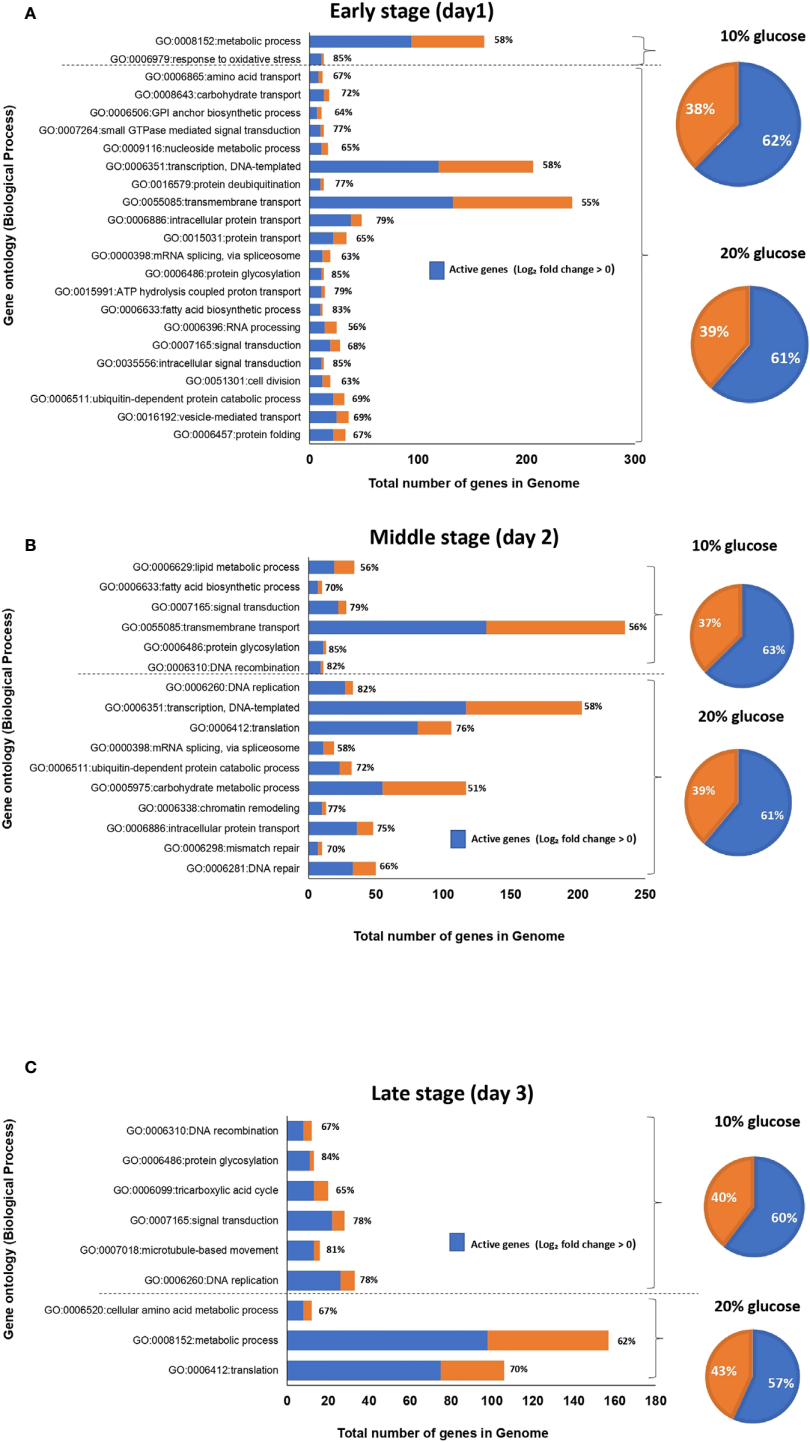
Graphical representation of enriched gene ontology (GO) terms for the biological process of *B. bassiana* blastospores produced in liquid medium amended with 20% glucose compared to 10% glucose after one **(A)**, two **(B)**, and three **(C)** days of culture (FDR *p* < 0.25). The value in front of each bar represents the percentage of active genes (log2-FC > 0), in the set of genes associated with each GO-term. The pie chart illustrates the percentage of active genes (in blue) in 20% or 10% glucose treatment considering all enriched GO-terms for each day of fermentation (see also **Supplemental Table S2**).

To characterize the set of genes significantly upregulated in blastospores cultivated in 20% and 10% glucose-amended media, GO-term gene set enrichment analysis using GSEA was utilized (Subramanian et al., 2005). A clear difference was found between GO-terms enriched in blastospores grown in 20% glucose medium compared to fungus grown in 10% glucose across three days of cultivation (**Figure 3**, **Supplemental Table S2**). On day 1 of culture, 53 GO terms were significantly enriched (FDR adjusted *p* < 0.25) in blastospores grown in 20% glucose medium. Of these 53 terms, 21 were assigned to biological process (Fig 3A), 8 to cellular components, and 24 to molecular function (**Supplemental Table S2**). Also on day 1, blastospores grown in 10% glucose medium had 12 GO-terms enriched, 2 in biological process category: response to oxidative stress (GO:0006979) and metabolic process (GO:0008152), and 8 in molecular functions category (**Figure 3A**, **Supplemental Table S2**). On day 2 of culture, 33 enriched GO-terms were found for blastospores grown in 20% glucose medium. Of these 33 terms, 11 were assigned to biological process, 6 to cellular components, and 16 to molecular functions and division (GO:0006310, GO:0006260, GO:0006412, GO:0006351) (**Figure 3**, **Supplemental Table S2**). Likewise, on day 2 for blastospores grown in 10% glucose medium 14 enriched GO-terms were found; 5 were assigned to biological processes, 3 to cellular components, and 6 to molecular functions (**Figure 3B** **;** **Supplemental Table S2**). These results agree with the laboratory observations of peak blastospore production on day 2 in both glucose-amended media. However, this peak is significantly higher for fungus grown under 20% glucose (**Figure 1A**). On day 3, blastospores grown in 20% glucose medium 22 enriched GO-terms were found; 3 were assigned to biological process (**Figure 3C**), 3 to cellular component, and 16 to molecular function (**Supplemental Table S2**). For blastospores grown in 10% glucose medium 6 GO-terms enriched among the biological process (**Figure 3C**) associated mainly with cellular growth/germination (GO:0006260, GO:0006310) were found. Additionally, 2 GO-terms assigned to cellular components and 9 to molecular function (**Supplemental Table S2**) were also identified.

The majority of GO-terms assigned to biological process: intracellular protein transport (GO:0006886), transcription, DNA-templated (GO:0006351), ubiquitin-dependent protein catabolic process (GO:0006511) were all enriched in blastospores grown in the high glucose medium at days 1 and 2, as well as for GO-term translation (GO:0006412) at days 2 and 3 compared to 10% glucose-amended medium (**Figure 3C**). These results indicate higher cell activity related to signal transduction, transmembrane transport, cell division, and transcription in blastospores grown with 20% glucose medium compared to those produced in the 10% glucose–amended medium.

#### Differentially Expressed Genes

**Figure 4:**
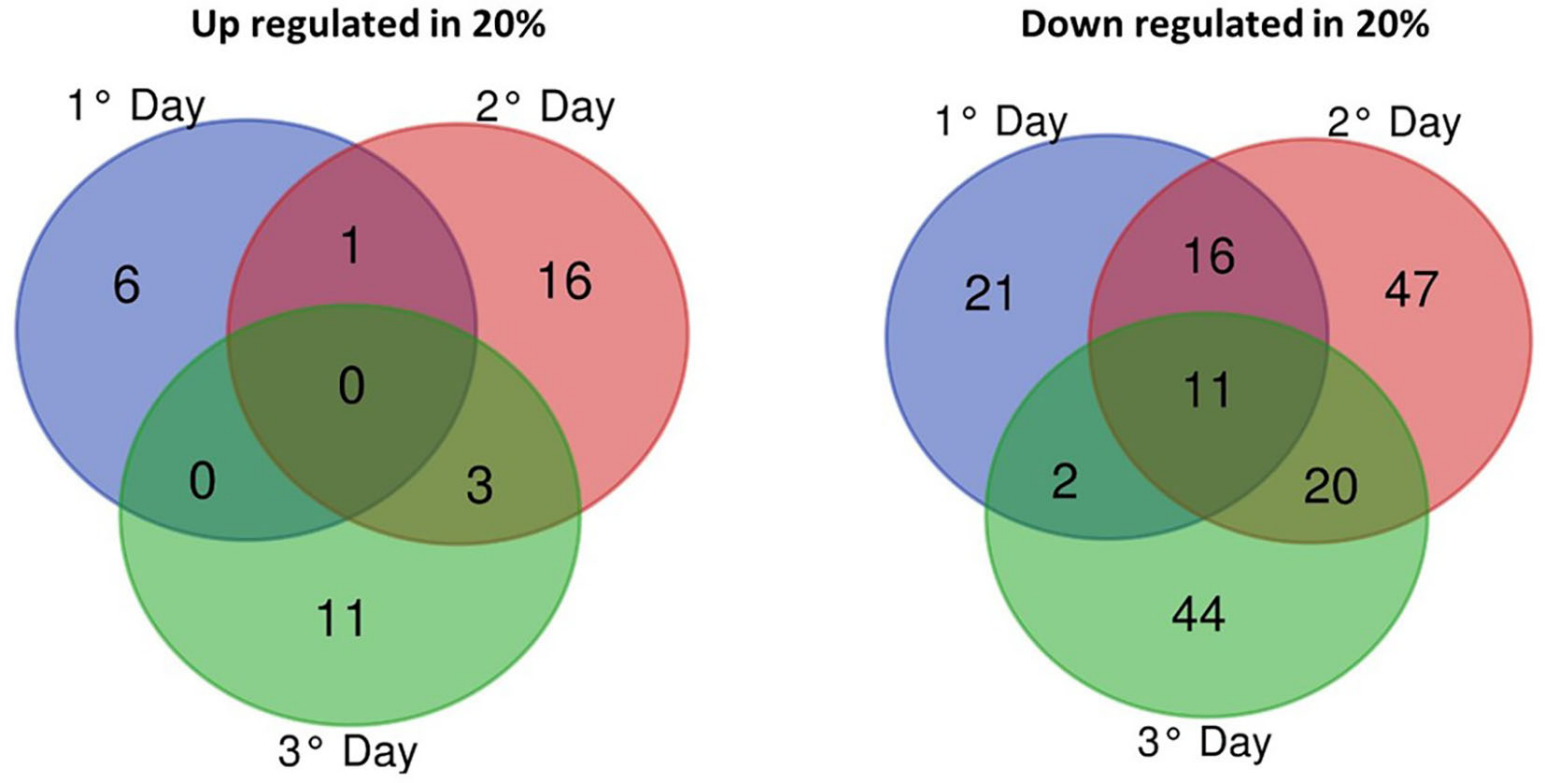
Venn diagram of differentially-expressed genes (log2-fold > 2 (upregulated) or < -2 (downregulated) of *B. bassiana* grown in liquid cultures supplemented with 20% vs. 10% glucose on days 1, 2, and 3 of fermentation. The cutoff used was false-discovery rate adjusted *p*-value less than 0.005 (FDR *p* < 0.005). DEGs, statistically significantly differentially-expressed genes (see also **Supplemental Table S3**).

From 11,861 genes annotated in the *B. bassiana* reference genome, 198 (1.66% of genome) genes were differentially expressed (FDR adjusted *p* < 0.005, log2-FC > 2 or < -2) between blastospores grown in medium amended with 10% and 20% glucose during three cultivation days. Most of the differentially expressed genes (DEGs) were downregulated in blastospores grown under 20% glucose representing a total of 161 genes (81% of the DEG) over 3 days of cultivation. Of these, 11 common genes were downregulated in all three days (**Figure 4**), with 7 encoding for “hypothetical proteins”, 1 for “trypsin” (BBAD15_g10782), 1 for “putative endo-1,3(4)-beta-glucanase” (BBAD15_g10674), and 1 for “Kinesin light chain” 4 (BBAD15_g12537) (**Supplemental Table S3**). Day 2 of cultivation corresponded to the highest number of downregulated genes (n=94), whereas day 1 showed the lowest number (n=50). Analyzing all time points of fermentation, a total of 37 genes (19% of the DEGs) were found to be upregulated in blastospores grown in the 20% glucose medium at least in one culture day. Day 2 of cultivation exhibited the highest number of upregulated genes (n=20), whereas, on day 1, there were only 7 upregulated genes (**Supplemental Table S3**). Moreover, a total of 37 genes (19% of the DEGs) were found to be upregulated in blastospores grown in the 20% glucose medium at least in one culture day.

**Figure 5:**
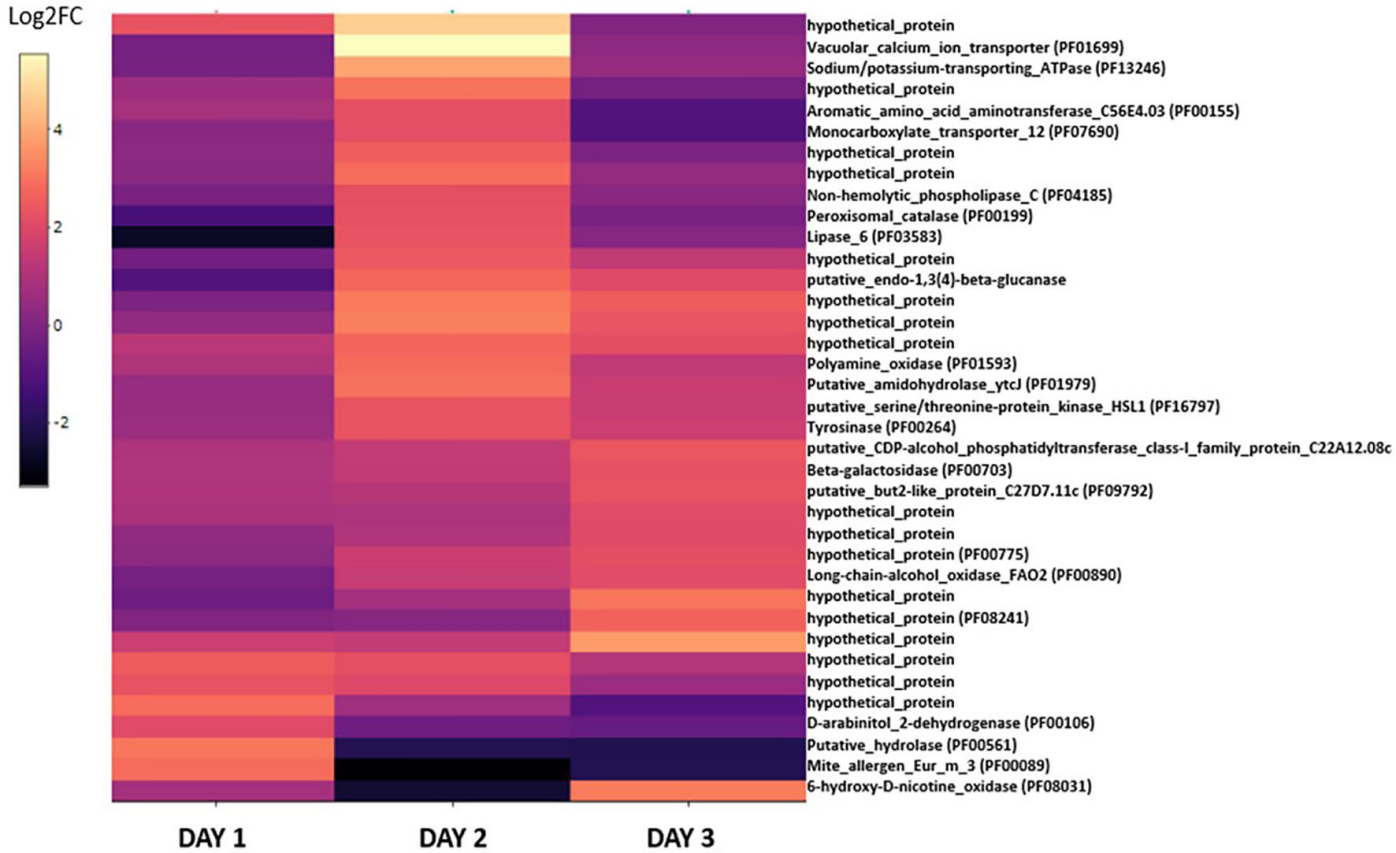
The heatmap displays the log_2_-fold changes of DEGs in the *B. bassiana* cultures grown under 20% glucose compared to 10% glucose-amended cultures. DEGs for at least one culture day (log2-FC > 2 (upregulated), FDR *p* < 0.005). In parentheses, the protein family code (pfam) provided when properly identified. Unknown proteins are assigned to “hypothetical proteins” (see also **Supplemental Table S3**).

The 37 genes upregulated in blastospores cultivated in the medium with 20% glucose were plotted in a heatmap to investigate these genes’ dynamism during the three days of cultivation (**Figure 5**, **Supplemental Table S3**). This process identified 17 genes assigned to hypothetical proteins with little or none annotated information available. On day 1, few genes were found upregulated and were represented mainly by enzymes such as hydrolase (PF00561), trypsins (PF00089), dehydrogenase (PF00106), and an oxidase (PF08031) (**Figure 5**, **Supplemental Table S3**). However, on day 2, a more significant change in the expression of genes related to nutrient transport (PF07690), oxidoreductase (PF01593), catalase (PF00199), and other enzymes such as lipase (PF03583) and tyrosinase (PF00264) were observed. Many upregulated genes found on day 2 were then down-regulated on day 3, and new groups of genes related to oxidase (PF00890), beta-galactosidase (PF00703), and hypothetical proteins were upregulated. Interestingly, the vacuolar calcium ion transporter (PF01699) and the sodium/potassium transporting ATPase (PF13246) were both strongly upregulated on day 2 when fungal cultures were grown with 20% glucose. This suggests blastospores on day 1 of fermentation relied on these proteins to maintain cation homeostasis through these cations’ active transport.

### Comparisons Between Culture Days Within 20% or 10% Glucose Medium

To identify the primary biological processes involved in the rapid blastopores growth in 20% glucose medium, GO terms enriched on day 2 in relation to day 1 and day were contrasted with those found in blastospores population grown in 10%-glucose medium.

#### Gene-Set Enrichment Analysis

**Figure 6:**
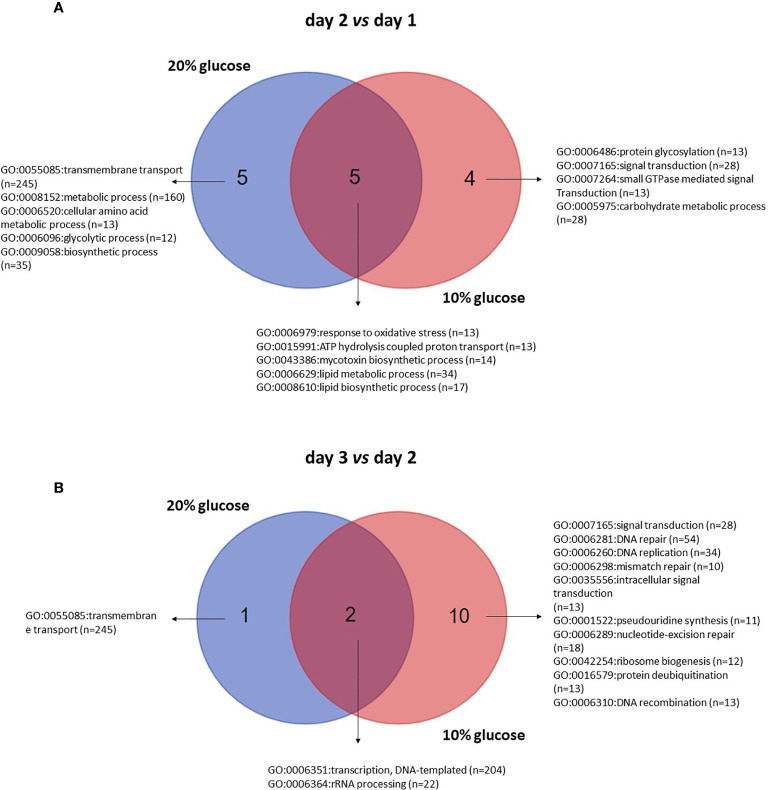
Venn diagram of enriched gene ontology (GO) terms for biological processes of blastospores of *B. bassiana* produced in medium amended with 20% or 10% glucose comparing day 2 with day 1 **(A)** and day 3 with day 2 **(B)** within each glucose concentration tested. FDR *p* < 0.25 (see also **Supplemental Table S4**).

Comparing day 2 with day 1, 4 and 5 exclusive GO-terms assigned to biological processes enriched in blastospores grown in 10% and 20% glucose medium, respectively, and 5 common enriched GO-terms (**Figure 6A**, **Supplemental Table S2**). On day 2, 53 GO-terms were significantly (FDR *p* < 0.25) enriched in blastospores grown in 10% glucose medium; 9 biological processes, 4 cellular components, and 22 molecular functions (**Figure 6A**, **Supplemental Table S2**). Likewise, 32 GO-terms significantly (FDR *p* < 0.25) enriched were found in blastospores grown in 20% glucose medium, 5 biological processes, 1 cellular component, and 26 molecular functions (**Figure 6A**, **Supplemental Table S2**). The common enriched GO-terms on day 2 were assigned to biological processes such as lipidic metabolic process (GO:0006629), lipidic biosynthetic process (GO:0008610), mycotoxin biosynthetic process (GO:0043386), ATP hydrolysis coupled proton transport (GO:0015991), and response to oxidative stress (GO:0006096) (**Figure 6A**). These results suggest 5 biological processes occur in the exponential growth phase (day 2), regardless of the initial glucose concentration supplied in liquid cultures of *B. bassiana*.

Comparing day 3 with day 2, 10 exclusive GO-terms assigned to biological processes enriched in blastospores grown in 10%, 1 exclusive GO-term enriched in blastospores cultivated under 20% glucose, and 2 common enriched GO-terms (**Figure 6B**) were found. On day 3, 35 GO-terms were significantly (FDR *p* < 0.25) enriched in blastospores grown in 10% glucose medium; 12 biological processes, 4 cellular components, and 19 molecular functions (**Figure 6B**, **Supplemental Table S2**). Additionally, 13 GO-terms enriched in blastospores grown in the 20% glucose medium; 3 biological processes (**Figure 6B**), 2 cellular components, and 8 molecular functions (**Supplemental Table S2**). The common enriched GO-terms at day 3 were assigned to biological processes such as transcription, DNA-templated (GO:0006351), and rRNA processing (GO:0006364). The only exclusive GO-term assigned to biological processes enriched in blastospores cultivated with 20% glucose was transmembrane transport (GO:0055085). The most significant difference (*p* < 0.05) in blastospore yield between 10% and 20% glucose media was observed in the *in vivo* experiment on day 3 (**Figure 1A**). On day 3 of cultivation, the blastospore concentration was twice greater in 20% glucose than when grown in 10% glucose.

#### Differentially Expressed Genes

The most considerable difference in gene expression of blastospores grown in different glucose concentrations (10% or 20%) was observed on day 2 compared to day 1, regardless of the initial glucose titer. A total of 507 down-regulated genes and 195 upregulated genes were observed on day 2 compared to day 1 for fungus grown with 20% glucose, and 560 upregulated and 259 down-regulated on day 2 compared to day 1 for fungus grown with 10% glucose (**Supplemental Table S3**). Additionally, 371 common upregulated genes on day 2 compared to day 1 when blastospores were produced with either 10% or 20% glucose medium with a significant number (n=181) classified as “hypothetical proteins”. Also, 189 and 137 genes were upregulated at day 2 in 10% and 20% glucose medium, respectively (**Supplemental Table S3**). Comparing day 2 with day 3, 49 DEGs were found in 10% glucose medium, of which 37 were upregulated and 12 were down-regulated. Conversely, 73 DEGs were found in 20% glucose medium, of which 24 were upregulated and 49 downregulated. Only 4 genes were commonly upregulated on day 3 for both 10% and 20% glucose enriched cultures; 3 of them were classified as “hypothetical proteins” (BBAD15_g2461, BBAD15_g7457, BBAD15_g5885), and 1 (BBAD15_g4395) was an isoflavone reductase (**Supplemental Table S3**), which is involved in the pathway pterocarpan phytoalexin biosynthesis.

### Expression of Heat Shock Protein Genes and Key Genes Associated With Response to Stress Conditions and High Osmolarity Glycerol Pathway

**Figure 8:**
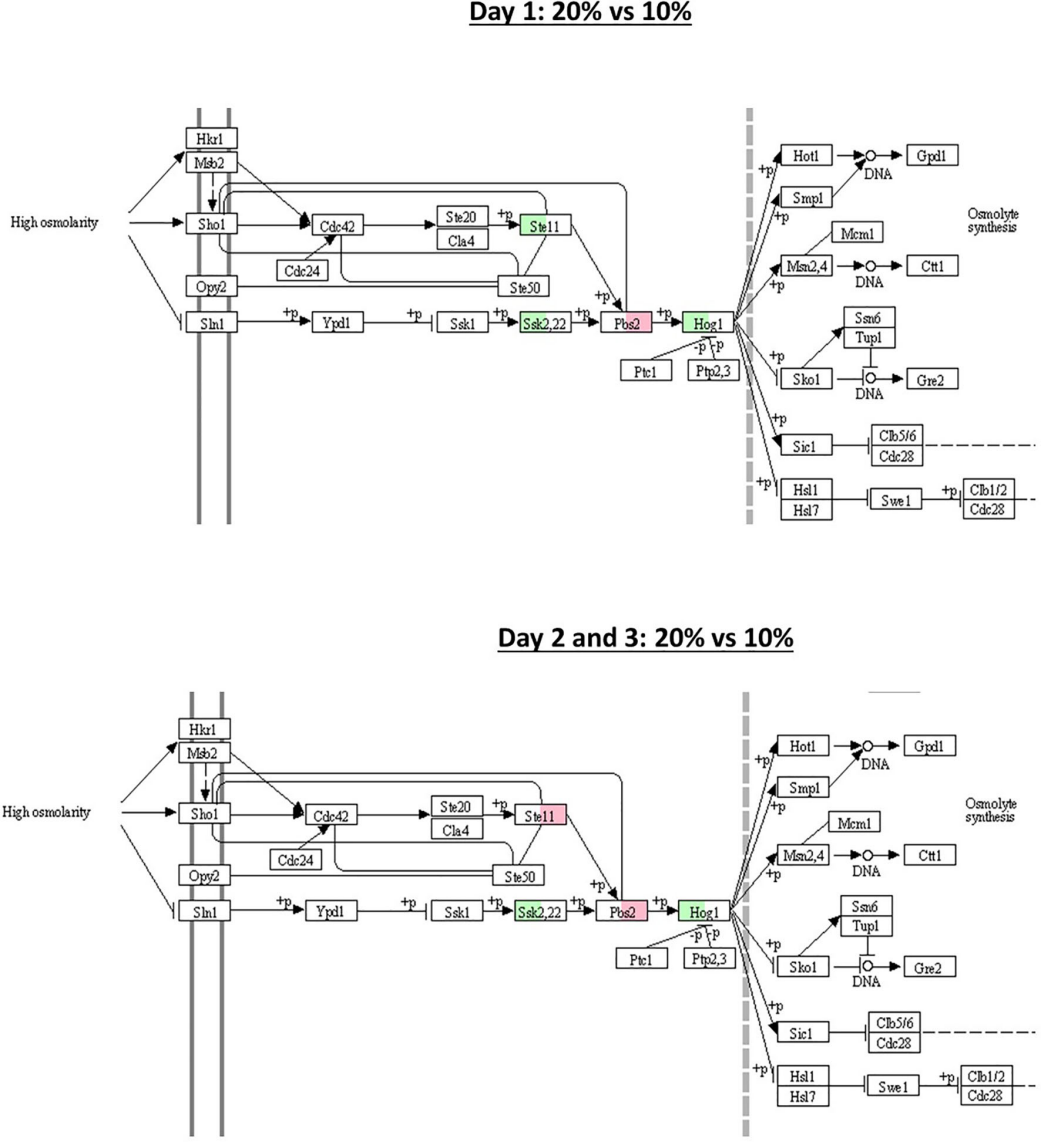
Schematic illustration of MAPK-signaling pathway with 5 selected genes (*Ssk1*, *Pbs2*, *Ste11*, *Hog1*, and *Ssk2*), known to be associated with response to osmotic stress. Green and red boxes indicate the gene is active (log2-FC > 0) in 20% and 10% glucose (log2-FC < 0) treatment, respectively (see also **Supplemental Table S4**). The pathway was obtained from the Kyoto Encyclopedia of Genes and Genomes pathway (KEGG) database.

In the *B. bassiana* GHA, 13 homologs HSP were identified out of the 14 HSP involved in stress responses, virulence, and cell wall integrity identified by Wang et al. (2020) in the *B. bassiana* ARSEF2860 genome. The expression of the 13 HSP70s homologous genes did not differ statistically between blastospores produced in 20% and 10% glucose, within each culture day (log2-FC > 2 < -2, FDR *p* < 0.005) (**Supplemental Table S4**). However, when fixing the glucose concentration and comparing the gene expression between culture days, 2 HSP70 homologs were found and are located in the cell cytoplasm: heat shock protein Ssb1 (BBAD15_g2252) and heat shock protein Ssz1 (BBAD15_g4682). Both were upregulated at day 1 compared to day 2 in blastospores grown in 20% and 10% glucose amended medium. Considering MAPK genes associated with the HOG pathway characterized by Liu et al. (2017), no statistical differences in the expression of homologs of *B. bassiana* D1-5 genes: *Ste11* (BBAD15_g9298), *Ssk2* (BBAD15_g1898), *Pbs2* (BBAD15_g4699), and *Hog1* (BBAD15_g6467), between blastospores produced in 20% and 10% glucose, within each culture day, nor between culture days when fixing glucose concentration (log2-FC > 2 < -2, FDR *p* < 0.005) (**Supplemental Tables S3** and **S4**). Although, the genes *Ssk2* and *Hog1* were found to be active (log2 -FC > 0) in 20% glucose medium in all culture days compared to 10% glucose (**Figure 8**). However, the homologs of the sensing protein Wsc1 (BBAD15_g1986), localized in the vacuoles and cell wall/membrane in *B. bassiana*, were upregulated on day 2 compared to day 1 in blastospores produced with both 20% and 10% glucose (**Supplemental Table S3**).

**Figure 7:**
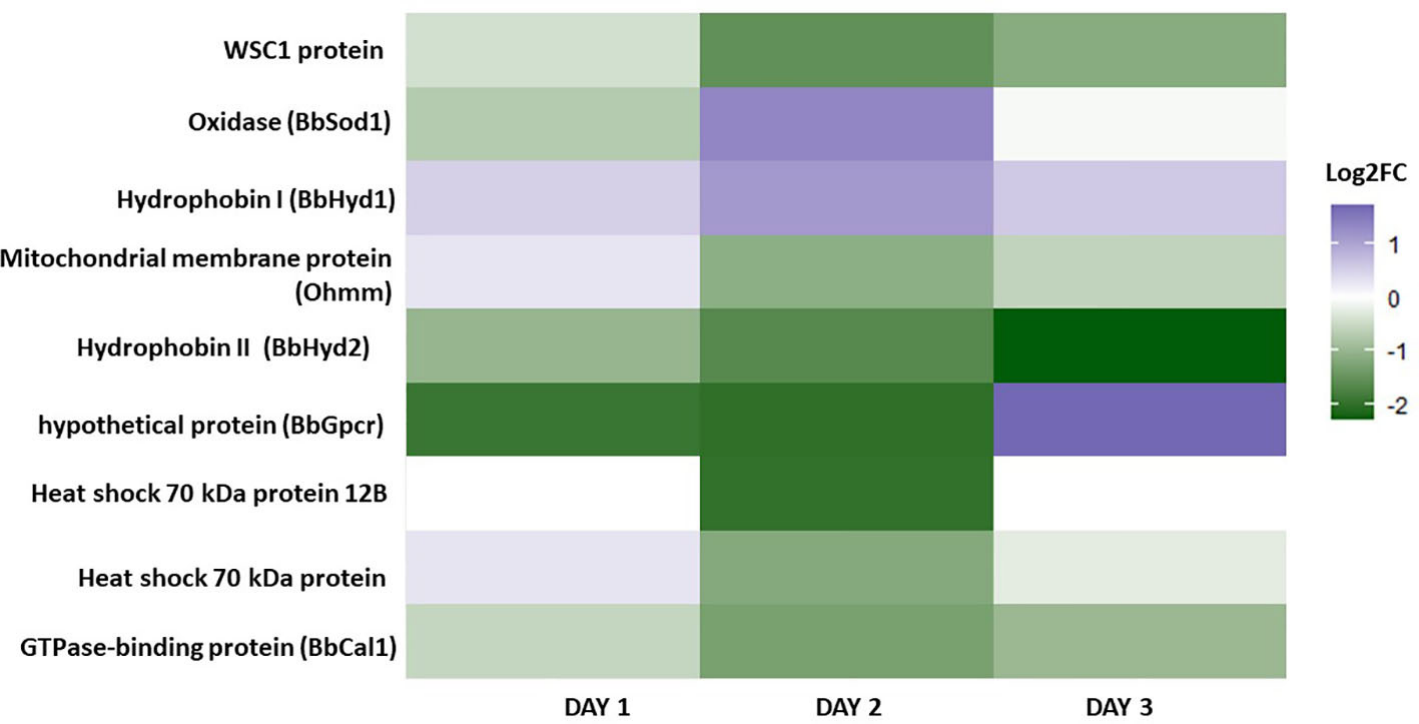
Heatmap depicting the gene expression of nine genes associated with osmotic stress in *B. bassiana* blastospores grown in liquid cultures supplemented with 20% vs. 10% glucose on days 1, 2, and 3 of fermentation (log2-FC >1 or < -1 in at least one of culture days) (see also **Supplemental Table S4**).

Because the media conditions suggest osmotic stress tolerance responses may be an essential factor in promoting this morphology’s development, 40 genes were reviewed involved in osmotic stress in *B. bassiana*, summarized in Ortiz-Urquiza and Keyhani (2016). The identification of 9 homologous genes were found in *B. bassiana* GHA involved in osmotic stress response in blastospores grown with 20% vs. 10% glucose on days 1, 2, and 3 of fermentation (log2-FC >1 or < -1 in at least one of culture days) (**Figure 7**, **Supplemental Table S4**). During the early phase of culture growth, at day 1 with 20% glucose, *B. bassiana* oxidative and osmotic related genes upregulated included the class I hydrophobin, *Hyd1* (BBAD15_g6903), and the mitochondrial membrane protein, Ohmn (BBAD15_g7953). These results inspired a closer look at the expression of these genes. *Hyd1* was highly expressed under all time points and both glucose rates, so a compiled list of the most expressed genes were made (**Supplemental Table S5**). All 6 genes were highly expressed, and represented the most expressed genes under all time points and both glucose rates. The genes with known function represent proteins associated with the cell wall, which is consistent with these proteins being the basic cell building blocks for these propagules.

In summary, by mapping key genes (*Ssk1*, *Pbs2*, *Ste11*, *Hog1*, and *Ssk2*) involved in the osmotic stress response in *B. bassiana* growing under 20% glucose (hyperosmotic medium) in relation to 10% glucose medium, we found implications of the HOG signaling pathway with upregulation of homologous genes *Ssk2* and *Hog1* for all time points of cultivation (**Figure 8**). These two genes are associated with cell osmoregulation leading to osmolyte synthesis and enhanced blastospore yield.

### Gene Expression of Key Blastospores-Related Genes *via* qRT-PCR

**Figure 9:**
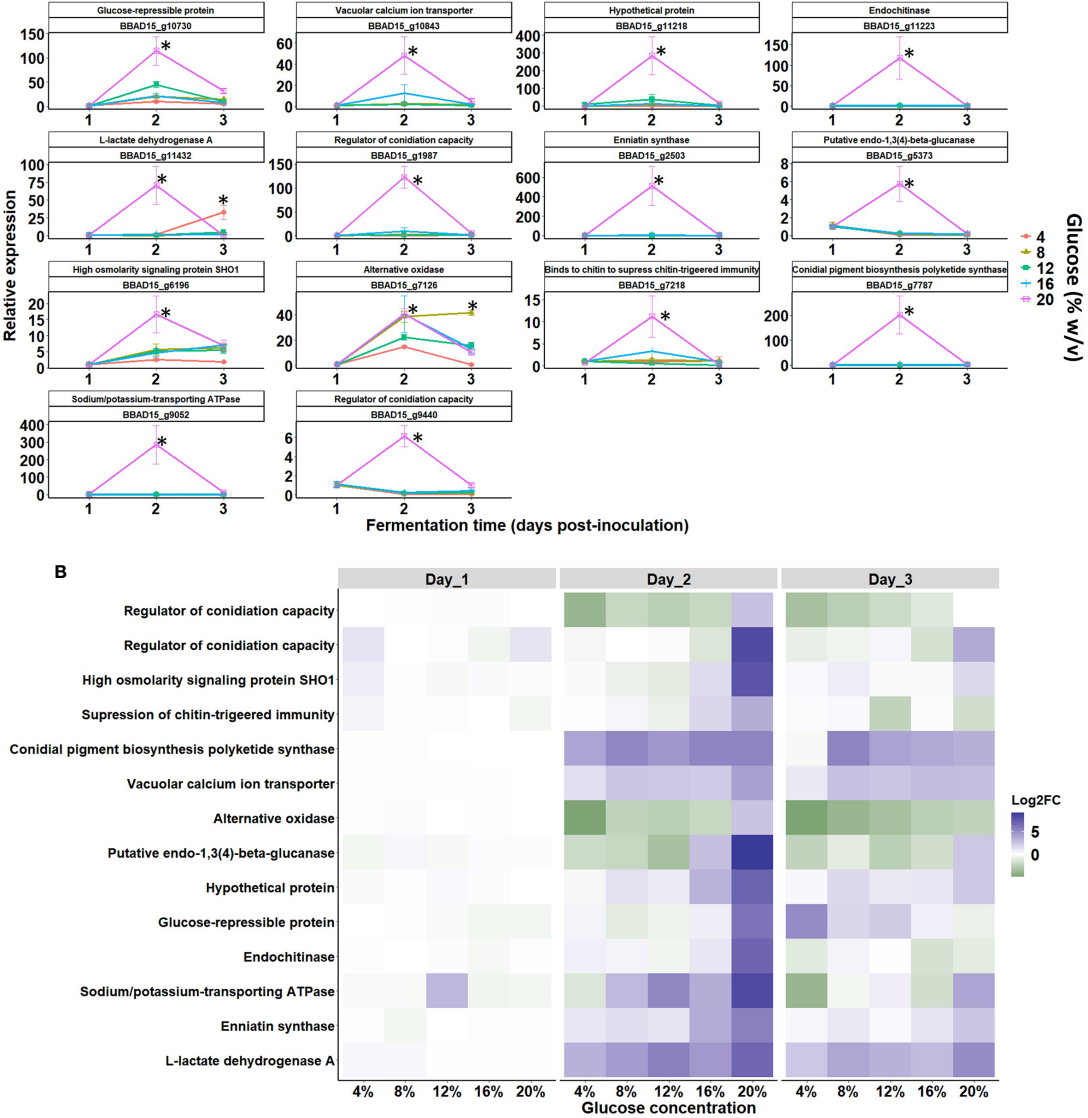
Gene expression of key blastospore-related genes *via* qPCR during growth stages of *B. bassiana* in response to an increased glucose gradient. **(A)** Temporal elicitation of selected *B. bassiana* genes (expressed in fold change values normalized by the reference actin gene *via* ΔΔCt method) during the blastospore growth stages: early (day 1), middle (day 2), and late (day 3) responses under liquid fermentation (mean ± standard error). Significant differences between glucose concentrations within each growth phase are indicated by asterisks (*p* < 0.05*) according to ANOVA. **(B)** Heatmap shows gene expression patterns at 1, 2, and 3 days post-inoculation and mean log_2_ fold change values from three biological replicates and two technical replicates. The log2-FC values and the color scale are shown at the right of the heat map, where blue indicates upregulation, while green indicates downregulation.

The expression of key genes involved in oxidative/respiration, carbon catabolite, cell wall synthesis, and osmoregulation pathways were tested via qRT-PCR. This was used to identify the essential fungal genes needed during blastospore growth under increased glucose rates on different fermentation days (**Figure 9**). The most pronounced gene expression, down- or upregulated, was seen at day 2 of cultivation, which coincided with the intense metabolic activity (catabolism and anabolism) during log-growth phase of *B. bassiana* blastospores. Notably, the expression level peak of all genes analyzed through qRT-PCR occurred with the growth peak of blastospores grown in 20% glucose (ANOVA, *p* < 0.05), which was the highest osmotic pressure simulated in this study (**Figure 9A**). Of particular interest, the gene expression profile showed strongly upregulated genes related to cell growth and cellular multiplication from day 2 to day 3 of development, compared to day 1, where genes were weakly expressed (**Figure 9B**). In this sense, all genes were highly upregulated after 2 days of cultivation in 20% glucose medium. More interestingly, the transcript levels of an osmosensor gene (BBAD15_g6196) were significantly upregulated at 20% glucose-enriched medium with a peak at day 2 and was maintained until day 3 of cultivation. Osmosensor gene expression levels initiated with an 8% glucose-amended medium and then continued to increase with the glucose gradient up to 20% (**Figure 9B**).

Furthermore, a high expression of two genes involved in regulating conidiation capacity was recorded in cultures grown with 20% glucose on day 2 compared to cultures grown at lower glucose levels (≤ 16% glucose) (**Figure 9B**). On day 3, the activity of endochitinase, beta-glucanase, and regulatory conidiation encoding genes decreased. Finally, analysis of gene BBAD15_g7126, which encodes for alternative oxidase (AOX), revealed a strong upregulation at all five glucose titers at day 2 and 3 of cultivation. Interestingly, gene regulation was slight or null on day 1 of cultivation, but on day 2, all genes were mostly upregulated with pronounced induction of all genes in blastospores grown under 20% glucose on day 2. During day 3, expression levels declined for most genes, irrespective of glucose concentration (**Figure 9B**).

## Discussion

Dimorphic growth characterized by the hyphae-blastospores transition in filamentous (anamorphic) entomopathogenic fungi is intriguing, primarily on how environmental and nutritional cues under certain liquid culture conditions trigger this transitional development into blastospores. This dimorphism of hyphae-blastospores in the host hemolymph is also critical for pathogen virulence (Wang et al., 2008; Ortiz-Urquiza and Keyhani, 2013). Previous studies have asserted that the coupling of high oxygenation and high glucose concentrations remarkably boost *in vitro* submerged multiplication of blastospores of *B. bassiana*, *Cordyceps javanica* and *Metarhizium robertsii* compared to cultures grown with low to moderate levels of glucose and aeration (Mascarin et al., 2015a; Mascarin et al., 2015b; Mascarin et al., 2016; Jaronski and Mascarin, 2017; Iwanicki et al., 2019; Iwanicki et al., 2020b). As a result, this combination of high osmotic pressure and oxygenation in the medium leads to an increased cell weight yield of a culture primarily composed of blastospores in 2 to 3 days of cultivation. In this regard, our results sheds light on the metabolic pathways linked to blastospore formation in highly aerated, glucose-rich cultures induced by oxidative and osmotic stresses that result in increased blastospore yield of *B. bassiana*. A high specific growth rate accompanied this improved blastospore yield, revealing an essential role of hyperosmolarity imposed by high glucose rate on the accelerated growth kinetics of blastospores, mainly represented by smaller (shrinkage) cells compared to those produced with low titers of glucose (≤10%), as noted by Mascarin et al. (2015b). The underlying molecular mechanisms involved with intense transmembrane transport, intracellular carbohydrate metabolism, intense antioxidant activity due to oxidative stress, enriched HOG-signaling pathway in response to osmotic stress, and DNA replication/transcription are boosted in day 2 with 20% glucose. This is the period with a distinct log-growth phase reflected in higher numbers of blastospores compared to cultures grown in 10% glucose. Future efforts to identify the underlying molecular mechanisms of cell shrinkage, osmoregulation, and cell cycle machinery should be related to kinetic growth dynamics and the multiplication of blastospores.

Our results indicate that the culture condition responses on day 1 were more pronounced in blastopores grown in a hyperosmotic medium amended with 20% than with 10% glucose. Although no differences in blastospore yield were observed on day 1 cultivated in 10% and 20% glucose media, our results showed a high number of enriched biological processes in blastopores grown in 20% glucose. The processes are associated with sensing and signal transduction, transport of protons against an electrochemical gradient using energy from ATP hydrolysis, proteins, amino acids, carbohydrate transport, and fatty acids biosynthesis. To maintain the shape and redox balance in the cytosol for optimal functioning of biochemical reactions in hyperosmotic conditions, yeast and other filamentous fungi use signal transduction process to respond rapidly by increasing intracellular solute concentrations, such as free amino acids, sugars, and fatty acids (Cliquet and Jackson, 1999; Westfall et al., 2004; Ding et al., 2019). Consequently, the rapid increase in solute concentration in the cytosol requires high ATP consumption by cells, which does not contribute to biomass synthesis (Varela et al., 2004). In hypersaline conditions, the halophilic fungus *Aspergillus montevidensis* accumulate fatty acids, amino acids, and soluble sugars to keep osmotic balance (Ding et al., 2019). Therefore, our findings indicate the 20% glucose medium triggers a rapid metabolic response of blastospores to deal with the imbalance in redox state by exchanging ions and accumulating solutes as observed by (Westfall et al. (2004) and Salmerón-Santiago et al. (2011) regarding fungal stresses.

Although we determined blastospores cultivated on high glucose (20%) concentration activate several more metabolic responses than those grown on 10% glucose on the first day of culture, we did not observe statistically significant gene expression differences (log2-FC > 2 < -2, FDR *p* < 0.005) among several MAPK homologous to *B. bassiana* ARSEF 2860 (Liu et al., 2017) associated with the HOG pathway. However, some individual genes were upregulated when using lower thresholds in the 20% glucose medium, as identified in **Figure 8**. In this sense, we hypothesized blastospores grown in 10% and 20% glucose medium are both under osmotic stress and activate genes associated with the HOG pathway at similar intensity.

Besides MAPK proteins associated with the HOG pathway, other important groups of proteins involved in response to stress conditions are the heat shock proteins (Hsps). Here, we identified two Hsps homologs to *Ssb1* (BBAD15_g2252) and *Ssz1* (BBAD15_g4682) upregulated at day 1 compared to day 2 in blastospores grown in 20% and 10% glucose. In *B. bassiana* ARSEF 2860, *Ssz1* and *Ssb1* homologous play similar roles as *FgSsb* and *FgSsz* in *Fusarium graminearum* (Liu et al., 2017) to tolerate high osmolarity and heavy metal cations (Wang et al., 2020), while in *Magnaporthe oryzae*, homologous to HSP *Ssb1* (*MoSsb1*) and HSP *Ssz1* (*MoSsz1*) were shown to be crucial for cell wall integrity, pathogenicity, growth, and conidiation (Yang et al., 2018). Altogether, these results indicate *Hsp, Ssb1* and *Ssz1* may be associated with sensing osmotic stress in blastospores, especially on day 1, regardless of the two glucose concentrations tested. These *Hsp* were not differently expressed on day 2, regardless of glucose concentration, indicating blastospores successfully respond to external stimuli. However, on day 2, blastospores had upregulated a gene homologous to *Wsc1* (BBAD15_g1986) protein, regardless of glucose concentration. Tong et al. (2019) characterized the functionally of WSC domain-containing protein Wsc1 in *B. bassiana* describing it as responsible for sensing multiple stress cues upstream of the Hog1 signaling pathway. These findings indicate blastospores might require genes homologous to the Wsc1 protein gene to deal with osmotic stress.

Our results from RNAseq analyses showed a remarkable variation in gene expression along the timeline of the experiment. Time accounted for 75% of the data variation, according to PCA-plot. Higher differences in gene expressions were observed on day 2 compared to day 1 of growth, regardless of glucose concentration. Enriched biological processes associated with DNA activities were observed in blastospores cultivated in 20% glucose on day 2. This can be related to intense cell growth and multiplication, as evidenced by *in vitro* experiments; statistically higher blastospore yield was confirmed in 20% glucose than in 10% glucose. This increase in blastospore yield in 20% glucose medium was also coupled with high upregulation of vacuolar calcium ion transporter (PF01699) and the sodium/potassium transporting ATPase (PF13246), as evidenced by the transcriptome and qRT-PCR results. Those genes are involved in the maintenance of cell homeostasis and were among the top 10 upregulated genes at day 2 in 20% glucose compared to 10% glucose.

We found blastospores cultivated in 10% and 20% glucose increased the expression of genes associated with oxidative stress responses more at day 2 than day 1. Corroborating this, a homologous gene to the sensing protein Wsc1 (BBAD15_g1986), localized in *B. bassiana* vacuoles and cell wall/membrane, was strongly upregulated at day 2 compared to day 1 in blastospores grown in both glucose concentrations. The deletion of this gene in a *B. bassiana* mutant led to a significant elevation in cell sensitivity to high oxidation, osmotic stress, and metal cations (Tong et al., 2019). At a moderate level, oxidative stress is an essential prerequisite for the growth and metabolism of several microorganisms including cell wall biosynthesis, cell proliferation, and morphogenesis (Nunes et al., 2005; Song et al., 2013; Iwanicki et al., 2020a). However, at high levels, free radicals’ accumulation with high reactivity such as superoxide, oxygen radicals, and hydroxyl, becomes harmful to the cells’ components. To cope with oxidative stress, blastospores activate enzymatic defense mechanisms by expressing enzymes such as superoxide dismutase, catalase, peroxidases, and oxidases. Several of these enzymes were upregulated at day 2. Moreover, we associated the response to oxidative stress as an essential component to boost blastospore production regardless of glucose concentration (Mascarin et al., 2015b; Iwanicki et al., 2020a). To some extent, the medium’s osmotic stress mimics insect hemolymph, the natural environment conducive for blastospores formation, and mediates the HOG signaling pathway enabling cell osmoregulation and water homeostasis (Wang et al., 2008; Liu et al., 2017).

Filamentous fungal insect pathogens like *B. bassiana* usually grow by hyphal extension outside the host and propagate by yeast-like budding only after entry into the host hemocoel subsequent to cuticular penetration. This allows the fungus to rapidly colonize the host hemocoel and evade the host’s immune responses. This dramatic change in morphology (phenotype) requires a dimorphic switch to regulate the hundreds of genes required for the transition. Since both aerial conidiation and dimorphic transition were closely linked to the transcriptional expression of the developmental activator genes (Zhang et al., 2019), it was hypothesized like aerial conidiation, the submerged dimorphic transition required for fungal virulence could be a process of asexual development to be primarily governed by the central pathway in *B. bassiana* and other filamentous fungal insect pathogens. These same authors unveiled *BrlA* and *AbaA* genes served as master regulators of both aerial conidiation and submerged blastospore production (dimorphic transition) (Zhang et al., 2019). In agreement with these findings, our qRT-PCR data revealed an increased transcript expression of genes responsible for the regulation of conidiation capacity (BBAD15_g9440 and BBAD15_g1987) in *B. bassiana* grown in liquid culture. This was mainly induced in blastospores produced in 20% glucose on day 2, thus hinting at their involvement in blastospores and conidia propagation. Further studies correlating the *BrlA* and *AbaA* genes with *B. bassiana* blastospore *in vitro* growth under high glucose titers should be addressed.

Carbon Catabolite Repression (CCR) is an important mechanism allowing preferential utilization of an energy-efficient and available carbon source over relatively less accessible carbon sources. This mechanism helps fungi to obtain the maximum amount of glucose for the energy invested. Fungi assimilate glucose and highly favorable sugars before switching to less suitable carbon sources such as organic acids and alcohols (Adnan et al., 2017). A glucose-repressible protein (BBAD15_g10730) examined in our qRT-PCR study was expressed in all glucose-amended *B. bassiana* cultures, but it was highly upregulated across all time points when the fungus was grown with 20% glucose. This result implies the catabolite repression pathway is strongly activated under high glucose titers required for increased and rapid production yields of *B. bassiana* blastospores in submerged liquid culture. Catabolite repression occurs at high glucose concentrations and is seen as the repression of synthesis of mitochondrial and other enzymes. Under these conditions, glucose is metabolized rapidly by glycolysis and considerable fermentation (anaerobic energy metabolism) occurs even under aerobic conditions. In other words, the NADH generated during glycolysis is oxidized by fermentation rather than by respiration, even though sufficient oxygen may be present. In this sense, the gene encoding for L-lactate dehydrogenase (BBAD15_g11432) analyzed by qRT-PCR was remarkably upregulated in blastospores cultured with 20% glucose by day 2, indicating the production of NAD+ (oxidized state), utilization of carbon sources, and maintenance of redox homeostasis during blastospores multiplication. The high metabolic activity of fungal blastospores cultivated with higher titers of glucose, in the range of 8-20%, increased up to day 3 and reflected the upregulation of alternative oxidase (AOX). The alternative oxidase (AOX) is a protein localized on the matrix side of the inner mitochondrial membrane. It plays a vital role in controlling reactive oxygen species (ROS), metabolic homeostasis, cellular energy demand, redox state, and stress response in fungal cells (Tian et al., 2020). Thus, AOX expression, mainly for high metabolic activity in cultures with higher glucose titers, helps the fungus cope with oxidative and osmotic stresses during fungal growth in liquid culture. AOX genes have been closely associated with fungal pathogenesis, morphogenesis, stress signaling, and drug resistance (Tian et al., 2020).

One interesting observation was hydrophobin *Hyd1* as one of the highest expressed genes during the propagule production. Hydrophobins are involved in fungal spore coat rodlet layer assembly, consistent with it being a key member of the cell wall. Previous studies have suggested *Hyd1* is not found in blastospores based on protein extraction (Holder and Keyhani, 2005) and immunostaining (Zhang et al., 2011), which raises the possibility that these might be mischaracterized as blastospores and are actually “submerged conidia” (Bidochka et al., 1987). It is tempting to conclude these are “submerged conidia” based on the amount of hydrophobin present (*Hyd2* is also relatively highly expressed, although much less than *Hyd1* [data not shown]), and this rodlet layer is a hallmark of conidiogenic propagules (Zhang et al., 2011). In addition to the rodlet layer, the main distinguishing features of submerged conidia are size and shape (spherical 3-4 um), and germination speed (Bidochka et al., 1987). Blastospores are typically ellipsoidal and larger (~6 µm), germinate faster, and lack a rodlet layer (Bidochka et al., 1987). In this study, the cells produced in 10% glucose appear more similar to the blastospore description (**Figure 1D**). In comparison, the cells grown in 20% glucose are smaller and more spherical (**Figure 1D**), as previously documented by Mascarin et al. (2015b). It is important to note *Hyd1* was one of the top five genes expressed under all time points and treatments during this experiment. Additionally, the 10% and 20% glucose-grown cells likely contain significant and similar amounts of hydrophobin 1. One possibility is hydrophobin 1 was not accessible during the previous attempts to observe it in blastospores (Holder and Keyhani, 2005; Zhang et al., 2011). If hydrophobin 1 is found in blastospores, it should be expressed in the haemocoel during the infection process, which it has been observed in previous transcriptomic studies (Cho et al., 2007; Dong et al., 2017). The *Hyd1* promoter has even been used to deliver an insecticidal toxin during the infection process (Wang et al., 2013). Interestingly, a *Hyd1* strain knockout had similar virulence when injected directly into the haemocoel (Zhang et al., 2011). Alternatively, if these are concluded to be a form of blastospore, it suggests size and shape are not useful to cell types indicators. It would also indicates that blastospores or another cell type containing hydrophobin 1 is expressed in the haemocoel during the infection process. Additional studies are needed to determine if these propagules should be considered blastospores, submerged conidia, or a different morphology. It also highlights the utility of transcriptomics in understanding the differences in morphologies when applied to a culture pushed to one predominant morphology.

Finally, by mapping key genes involved in the osmotic stress response in *B. bassiana* growing under a hyperosmotic environment (**Figure 8**), we found implications of the HOG signaling pathway with upregulation of homologous genes *Ssk2* and *Hog1*. We hypothesize these two genes are associated with cell osmoregulation, leading to osmolyte synthesis and enhanced blastospore yield. The *Hog1* has also been linked to regulating environmental stresses in *B. bassiana* (Zhang et al., 2009). Deletion of the *Hog1* gene also reduced *Hyd1* expression by 46x, suggesting strong regulation of *Hyd1 via Hog1* (Zhang et al., 2009).

## Conclusions

Our findings pave the way towards improving the fermentation process for *B. bassiana* and generates much-needed new knowledge on the primary metabolic processes and gene expression signatures involved in *B. bassiana* grown under osmotic and oxidative conditions with improved blastospore yields. Considering other hypocrealean entomopathogenic fungi, such as *Cordyceps* (formerly *Isaria*) and *Metarhizium*, have their blastospore production enhanced when grown with highly aerated and hyperosmotic liquid cultures, future comparative transcriptome studies should confirm the universal genetic regulations underlying this intriguing phenomenon. We envision the transcriptome tool allied with metabolomics will help improve the overall industrial production of blastospores and their metabolites for agricultural, pharmaceutical, and biotechnological purposes.

## Data Availability Statement

The original contributions presented in the study are publicly available. This data can be found here: https://www.ncbi.nlm.nih.gov/geo/query/acc.cgi?acc=GSE163673.

## Author Contributions

CD, GM, and JLR conceived the experiments. GM, JLR, and CD conducted the experiments. All authors analyzed the results, prepared a manuscript draft, and edited the manuscript. All authors revised the manuscript for technical and scientific accuracy. JLR and CD acquired funding. CD and GM supervised the project. All authors contributed to the article and approved the submitted version.

## Funding

This research is supported by the USDA ARS Project #510-22410-023-00-D.

## Conflict of Interest

Author GMM was employed by Brazilian Agricultural Research Corporation.

The remaining authors declare that the research was conducted in the absence of any commercial or financial relationships that could be construed as a potential conflict of interest.

## Supplementary Material

Supplementary Table 1 Primer sequences used for qRT-PCR gene expression analyses.

Supplementary Table 2 Enriched Gene Ontology (GO), terms in *Beauveria bassiana* blastospores (between 20% and 10% within each culture day, and between culture days within each glucose concentration) resulted from Gene set enrichment analysis.

Supplementary Table 3 Dataset with significantly differentially expressed genes between glucose concentrations within each culture day, and between culture days within each glucose concentration, based on log2-FC > 2 (upregulated) or < -2 (downregulated) with FDR at *p* < 0.005.

Supplementary Table 4 Homologous (*e*-value < 0.0001 and sequence similarity > 80%) to 14 Hsp70 sequences, four MAPK sequences, and one sequence (Wsc1) obtained from Liu et al. (2017); Wang et al. (2020), and Tong et al. (2019), respectively, and 40 related-genes to osmotic stress in *B. bassiana* (Ortiz-Urquiza and Keyhani, 2016).

Supplementary Table 5 The six highest expressed genes over all treatments based on total summed daily percentages.
