# Supplementary material for: Transcriptional Responses of Beauveria bassiana Blastospores Cultured Under Varying Glucose Concentrations
Source: Front Cell Infect Microbiol. 2021 Mar 24;11:644372. doi: 10.3389/fcimb.2021.644372 (PMC8024584; doi:10.3389/fcimb.2021.644372)
Supplement: Supplementary Table 1 — Primer sequences used for qRT-PCR gene expression analyses. [file Table_1.docx]

**Table S1.** Primer sequences used for qPCR gene expression analyses.

| Gene-contig ID | Forward Primer | Reverse Primer | Reference |
| --- | --- | --- | --- |
| BBAD15_g6196 | CCATGCTCAACATTGTCTGG | GACAGGGCAAAAGAGTCGAG | This study |
|  |  |  |  |
| BBAD15_g9440 | CTCACTGCACTCACCGAAAA | TCCACGATCTTCTTGCTCCT | This study |
|  |  |  |  |
| BBAD15_g1987 | GTCGTATATGCCCGACGAGT | TGGTCATCATCTCCGACAAA | This study |
|  |  |  |  |
| BBAD15_g7218 | TGGCTGGTACTACTGCGTTG | ACCACTGACCACACTTGCAG | This study |
|  |  |  |  |
| BBAD15_g7787 | ACCTTTGACACCCAGGACAG | CTTGACGTGCTGAAAGACCA | This study |
|  |  |  |  |
| BBAD15_g10843 | TCAGTAATGGCCACGTGAAA | GGATAGGCAGGAGGTTGACA | This study |
|  |  |  |  |
| BBAD15_g7126 | GGTGCACAGGGTGTCTTTTT | CCTCTTCGAGATAGCCAACG | This study |
|  |  |  |  |
| BBAD15_g5373 | CAGAGCTGGGTGCTGTCAC | CTGTGCAACGGCAAAAAGTA | This study |
|  |  |  |  |
| BBAD15_g11379 | GACCAACTTTGGTGGTGTCG | TCACCGAGCTTGAAGAGGTT | This study |
|  |  |  |  |
| BBAD15_g10730 | CCAAGGAGACCAACAAGGAA | CTCTCGTCAAGCTTGTCACC | This study |
|  |  |  |  |
| BBAD15_g9052 | TCTCTGCAAGTCGCTCAAGA | GAAGTTCTTCGGTGCTGAGG | This study |
|  |  |  |  |
| BBAD15_g11432 | ATCTCCGTGATTCCACAAGC | AGATTCTCCGGCTTTTTGGT | This study |
|  |  |  |  |
| BBAD15_g11223 | CAACTGTACGCCAATCATGG | CTCTCCGGGCTAGAAGGAGT | This study |
|  |  |  |  |
| BBAD15_g11218 | AGTATGTAGGCCGCGTCGTT | GGCCTGGTACACTGCGTTA | This study |
|  |  |  |  |
| Actin (HQ232398.1)* | GTCAAGTCATCACCATTGGC | GAGGAGCAATGATCTTGACC | Fan et al., 2016 |
|  |  |  |  |

*Reference gene for computing the relative expression level of key genes.
